# An *EGFP* Knock-in Zebrafish Experimental Model Used in Evaluation of the Amantadine Drug Safety During Early Cardiogenesis

**DOI:** 10.3389/fcvm.2022.839166

**Published:** 2022-04-05

**Authors:** Shi Ouyang, Wu-Ming Qin, Yu-Juan Niu, Yong-He Ding, Yun Deng

**Affiliations:** ^1^State Key Laboratory of Developmental Biology of Freshwater Fish, Hunan Normal University, Changsha, China; ^2^Laboratory of Zebrafish Genetics, College of Life Sciences, Hunan Normal University, Changsha, China; ^3^The Affiliated Hospital of Qingdao University, Qingdao University, Qingdao, China; ^4^The Biomedical Sciences Institute of Qingdao University (Qingdao Branch of SJTU Bio-X Institute), Qingdao University, Qingdao, China

**Keywords:** zebrafish model, *vmhc*, amantadine drug, congenital heart disease (CHD), cardiac development

## Abstract

**Background:**

Drug exposure during gestation or in prematurely born children represents a significant risk to congenital heart disease (CHD). Amantadine is an antiviral agent also effective in the treatment of Parkinson’s disease. However, while its potential side effects associated with tetralogy of fallot (ToF) and birth defects were implicated, its underlying etiologic mechanisms of action remain unknown. Here, we report teratogenic effects of amantadine drug during early cardiogenesis through developing a novel zebrafish (*Danio rerio*) knock-in (KI) animal model and explore the underlying mechanisms.

**Methods:**

Homologous recombination (HR) pathway triggered by CRISPR/Cas9 system was utilized to generate an enhanced green fluorescent protein (EGFP) KI zebrafish animal model. Dynamic fluorescence imaging coupled with a whole-mount *in-situ* hybridization (WISH) assay was employed to compare the spatial and temporal expression patterns of the EGFP reporter in the KI animal model with the KI-targeted endogenous gene. Heart morphology and EGFP expression dynamics in the KI animal models were monitored to assess cardiac side effects of different doses of amantadine hydrochloride. Expression of key genes required for myocardium differentiation and left–right (LR) asymmetry was analyzed using WISH and quantitative reverse transcription-PCR (RT-PCR).

**Results:**

A novel EGFP KI line targeted at the ventricular myosin heavy chain (*vmhc)* gene locus was successfully generated, in which EGFP reporter could faithfully recapitulate the endogenous expression dynamics of the ventricle chamber-specific expression of the *vmhc* gene. Amantadine drug treatment-induced ectopic expression of *vmhc* gene in the atrium and caused cardiac-looping or LR asymmetry defects to dose-dependently during early cardiogenesis, concomitant with dramatically reduced expression levels of key genes required for myocardium differentiation and LR asymmetry.

**Conclusion:**

We generated a novel zebrafish KI animal model in which EGFP reports the ventricle chamber-specific expression of *vmhc* gene dynamics that is useful to effectively assess drug safety on the cardiac morphology *in vivo*. Specifically, this study identified teratogenic effects of amantadine drug during early cardiogenesis dose dependent, which could be likely conveyed by inhibiting expression of key genes required for cardiac myocardium differentiation and LR asymmetry.

## Introduction

Congenital heart disease (CHD) is one of the most common types of birth defects which affect about 1% of infants born each year (1). The etiology of CHD is multifactorial, with both genetic and environmental factors such as exposure to teratogens playing important roles. Amantadine hydrochloride is an organic compound widely used to treat Parkinson’s disease, Friedreich’s ataxia, multiple sclerosis, and influenza A virus infection (2, 3). Previous studies reported the potential link of amantadine hydrochloride treatment at the dose of 100 mg/day during the first trimester of pregnancy to cardiac defects and tetralogy of fallot (ToF) in patients (4, 5). Additional reports in pregnant rats and rabbits also implicated the potential teratogenic and embryotoxic effects of amantadine hydrochloride at a dose equivalent to the estimated maximum dose used in humans (6, 7). Despite this observed link between amantadine hydrochloride treatment and subsequent toxicity, the overall mechanism of action remains unknown. Compound paracetamol and amantadine hydrochloride tablets (CPAAHTs) are used widely for the relief of symptoms caused by influenza and for the treatment of Parkinson’s disease that contains amantadine hydrochloride as a major ingredient. Whether exposure to overdosage of CPAAHTs could exert considerable side effects on heart development and/or cause birth defects is yet to be determined.

Zebrafish (*Danio rerio*), due to its high fecundity, rapid embryonic development, optical transparency, and highly conserved cardiac electrophysiology to humans, has emerged as a prolific animal model for studying drug toxicity and pathophysiology during early cardiogenesis *in vivo* (8–10). With the advent of CRISPR/Cas9-based genomic editing technology that enables efficient gene knock-out (KO) and KI at targeted gene loci (11–13), interaction studies between genes and environmental factors such as drug exposure have become more feasible. In zebrafish, double-strand breaks (DSBs) could be induced by CRISPR/Cas9 systems, which subsequently trigger organism repair mechanisms through nonhomology end joining (NHEJ) and HR pathways (12, 14). KI events can incur through either the NHEJ or the HR repair pathway. While the efficiency of NHEJ is much higher than HR, the HR-based targeted insertion is more precise than NHEJ.

Ventricle myosin heavy chain (*vmhc*) is a ventricle chamber-specifically expressed cardiac myosin gene that represents one of the earliest myocardial markers for revealing the ventricular cell lineages. Its atrium suppressed expression is mediated through the cardiac development regulators such as *nkx2.5* and *gata4* (15, 16).

Here, we firstly knocked in an EGFP reporter into the *vmhc* gene by inserting an exogenous *EGFP* reporter replacing the stop codon of the *vmhc* gene through the HR pathway using CRISPR/Cas9. Thus, we generated the so-called *vmhc^KI–EGFP^* stable KI line in which the EGFP could faithfully report the spatial and temporal expression patterns of the endogenous *vmhc* gene, without affecting the expression of its encoded vmhc protein. Next, we employed this *vmhc^KI–EGFP^* KI zebrafish animal model to assess the potential side effects of CPAAHTs during early cardiogenesis and investigated the underlying etiologic mechanisms.

## Materials and Methods

### Animals

Zebrafish (*Danio rerio*) (TU strain) was obtained from the China Zebrafish Resource Center (Wuhan, China) and maintained on a 14 h light/10 h dark cycle at 28.5°C. All the animal study procedures were performed in accordance with the Guide for the Care and Use of Laboratory Animals published by the US National Institute of Health. Animal study protocols were approved by the Institutional Animal Care and Use Committee of Hunan Normal University.

### Generation of *vmhc*^KI–EGFP^** Knock-in Fish

The *vmhc* KI fish line: *vmhc^KI–EGFP^* was generated by co-injection of single guide RNA (sgRNA), Cas9 protein, and the *vmhc*-P2A-EGFP donor vector into one-cell stage embryos.

The sgRNA targeted to the C-terminal of *vmhc* gene including the stop codon (GAUCAAGAGUAAGCUCAAGUGG) was *in vitro* transcribed using the MAXIscript™ T7 Transcription Kit (Invitrogen) according to the manufacturer’s instructions, followed by purification using the RNeasy Mini kit (Qiagen). Cas9 protein was purchased from TrueCut™ Cas9 Protein v2 (Invitrogen). In total, 40 ng sgRNA and 0.5 ng Cas9 protein complex were assembled by incubation for 10 min at 37°C and then was injected into one-cell stage embryos. Knockout efficiency of the sgRNA was determined by PCR and DNA gel analysis using genomic DNA isolated from injected embryos at 48 h postfertilization (hpf).

The *vmhc*-P2A-EGFP donor vector was constructed by cloning of the *vmhc* left and right homologous arm fragments into the *p*MCS1-P2A-EGFP-MCS2 vector. Briefly, a 2,218 bp left homologous arm fragment of the *vmhc* gene was PCR amplified with genomic DNA isolated from zebrafish tail fin using primers: left-F: (GGTCGACCACTCCCAGGTATGCTAAATGTTT) and left-R: (GGGATCCGACTCTTGATCATGTCCCTTCTGTA). The resultant PCR fragments were digested with *Sa*l I and *Bam*H I and cloned to the MCS1 site of the *p*MCS1-P2A-EGFP-MCS2 vector to generate *p*vmhc-left-P2A-EGFP-MCS2. Subsequently, to generate the final recombinant donor vector: *vmhc*-P2A-EGFP, a 1,926 bp right homologous arm fragment of the *vmhc* gene was PCR amplified using primers right-F: (GGCGGCCGCTGTGTCTCCGTTATGCTGAATT) and right-R: (GCTCGAGTTTGGCCGTTATAGTCCAGAAAC) and subcloned into the MCS2 sites of *p*vmhc-left-P2A-EGFP-MCS2 after digestion of Not I and Xho I. Correct clones of both the *vmhc* left and right homologous arms were further confirmed by Sanger sequencing.

Next, ∼1 nl solution containing 40 ng sgRNA, 400 ng donor recombinant vector, and 0.5 ng Cas9 protein was co-injected into one-cell stage embryos. Injected embryos with EGFP positive signals were raised to adulthood. Injected adult F0 fish were outcrossed with wide-type, and stable knock-in F1 fish with germline transmission were identified by positive EGFP signal and subsequent PCR validation using primers F1: TCTGTCTCATACTTCAGCCTCC, R1: GTCGTCCTTGAAGAAGATGGTG; F2: CCACAACGTCT ATATCATGGCC, R2: ATACACTCCATACATACACGCG, and Sanger sequencing.

### Western Blotting

Total protein was homogenized and extracted from 3 pooled embryos at 3 dpf using radioimmunoprecipitation assay (RIPA) solution (Beyotime Biotechnology, Shanghai, China) and was subjected to standard western blotting analysis. Briefly, extracted total protein was separated in 6% polyacrylamide gels, transferred onto nitrocellulose membrane, and subsequently incubated with primary and secondary antibodies. The primary antibody for anti-*vmhc* was obtained from proteintech (1:1,000, 22280-1-AP), and anti-tubulin was purchased from Affinity Biosciences (1:2,000, T0034). The secondary antibody was purchased from Affinity Biosciences (S0002). Quantification of relative protein expression was performed using Image J and student *t*-test was used to calculate the *P-value*.

### Drug Treatment

One-cell stage *vmhc^KI–EGFP^* embryos were incubated in the presence or absence of CPAAHTs (Gankang pharmaceutical, Wuhan, China) dissolved in E3 water (5 mM NaCl, 1 mM MgSO_4_, 1 mM KCl, and 1 mM CaCl_2_) at designated concentrations for 48 h. Cardiac morphology of both drug-treated and untreated control embryos were monitored under a fluorescence microscope (Leica, Berlin, Germany). To compare the EGFP signal intensity among different experimental groups, we used the same exposure time when the fluorescent images were captured. In total, 5–10 embryos were randomly chosen from each group for images capture. All the experiments were performed in triplicate.

### Whole-Mount *in-situ* Hybridization

Embryos with or without CPAAHTs treatment were randomly picked up and subjected to whole-mount *in-situ* hybridization (WISH) accordingly (17). Digoxigenin-labeled anti-sense RNA probes were generated from PCR products using T7 RNA polymerase or T3 RNA polymerase from the DIG-RNA labeling mix (Roche) according to the manufacturer’s instruction. The PCR primer sequences used for RNA probes synthesis are listed here: *nkx2.5*-ISH-F: CTTCCACTCCTTTCTCAGTGC, *nkx2.5*-ISH-R: TGCATGAGTAGTTCGAGTTGC; *tbx20*-ISH-F: GGCACTGAAATGATTATCACAAAG,

*tbx20-ISH-R*: CTTCTCCCAGAGTCTCTTCATCTC; *mef2ca*- ISH-F: GATCGCCCTCATCATCTTCAAC, *mef2ca*-ISH-R: ATG AGGTATTGATGGCAGACGG; *gata4*-ISH-F: CGAGTCCGG TTTCCTCCATA, *gata4*-ISH-R: TCGTGTCTGAATGCCCTCT T; *spaw*-ISH-F: CTGGGCAGCGTTAAATCAGTAG, *spaw*-ISH -R: CTCATCACCACTCCATCTCCTT; *lft2*-ISH-F: GATAAAC CACGCCAGAGTCAG, *lft2*-ISH-R: CGGAAGTTGATGAAA TGCTCCT; *pitx2*-ISH-F: CGCATTTTACTAGCCAGCAGT, *pi tx2*-ISH-R: ACTGGCAAGACTGGAGTTACA; *lft1*-ISH-F: ATGACTTCAGTCCGCGCCGCG, *lft1*-ISH-R: TTATACAACT GAAATATTGTC.

### Quantitative Reverse Transcription-PCR

In total, three pooled embryos with or without CPAAHTs treatment were randomly picked up for total RNA extraction using Trizol (Thermo Fisher Scientific, Waltham, MA, United States). About 1 μg total RNA was used for reverse transcription and cDNA synthesis using the RevertAid First Strand cDNA Synthesis Kit (ThermoFisher Scientific) according to the manufacturer’s instruction. Real-time quantitative PCR was run using the Computer Forensic Examiner (CFX) Connect Real-Time System (BIO-RAD, Hercules, CA, United States) in a total volume of 10 μl reaction solution containing 1 × SYBR Green Master Mix (Yeasen Biotechnology, Shanghai, China), 0.2 μM gene-specific primer pairs, and 1 μl cDNA template. A three-step PCR with a 60°C annealing temperature was used for all primers. Gene expression levels were normalized using the expression level of *ββ-actin* by –ΔΔCt (cycle threshold) values. All gene-specific primer sequences are listed here: *gata4*-F: GCCTATGTGAGCCCTAATATC, *gata4*-R: CCTCGCCCAGA TCATCAAA; *mef2ca*-F: AAAGTTCTGCTGAAATACACCGAG, *mef2ca*-R: CGTAGTTAGACTGAGGGATGGC; *tbx20*-F: GCA CTCATGTCAAGTGGGAA, *tbx20*-R: CGAGGTTTGGATGG CATGA; *spaw*-F: CACGCTGAACCGACCAGCAG, *spaw*-R: CAAAGTGAAGCTTGCTGTTCC; *pitx2*-F: TATCCGGACAT GTCGACTAGA, *pitx2*-R: CCTGTTGATTCCTCTCCCTTT; *lf t1*-F: CCTCAGAAAGACGGGTCAAA, *lft1*-R: CGCCTGGGT GACATCAAA; *lft2*-F: GCAAGTATCTGTCCATGCTGA, *lft2*-R: GTGGTGTCCGAGTACTTGATTT; *nkx2.5*-F: CTTCAGTGC TTCAGGCTTTTACGCG, *nkx2.5*-R: GCTCCGCATCATCCA GCTTCAGATC; *β-actin*-F: CGTGACATCAAGGAGAAG, *β-actin*-R: GAGTTGAAGGTGGTCTCAT.

### Statistical Analysis

Unpaired two-tailed Student’s *t*-tests were used for comparisons of two groups. The chi-squared test was used to determine the effects of different concentrations of CPAAHTs on cardiac looping during cardiogenesis. *P*-values of less than 0.05 were considered as statistically significant. All the statistical analyses were carried out using GraphPad Prism version 7.0 software.

## Results

### Generation of the *vmhc*^KI–EGFP^** Knock-in Zebrafish Line

**FIGURE 1:**
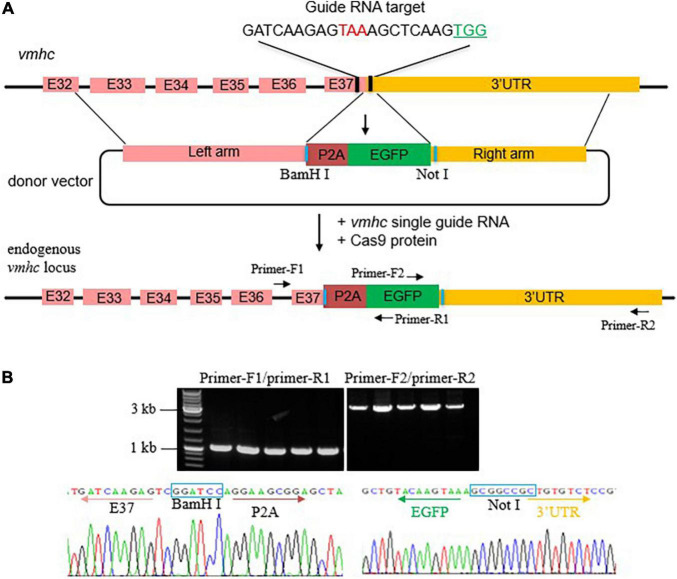
Generation of the *vmhc^KI–EGFP^* zebrafish KI line. **(A)** Schematic diagram of the KI strategy based on the HR pathway triggered by the CRISPR/Cas9 system. The TAA stop codon (in red) is included in the guide RNA target sequences. Protospacer adjacent motif (PAM) sequences are marked in green. The donor vector used for HR contains the left homologous arm, P2A-EGFP, and right homologous arm. Restriction enzymes sites used for left and right homologous arms cloning are marked in blue. **(B)** Correct integration of P2A-EGFP into the C-terminal of the endogenous *vmhc* locus was confirmed by genotyping PCR and Sanger sequencing in the F1 stable KI embryos.

To generate an EGFP KI fish in the *vmhc* gene, we first designed and synthesized a single guide RNA (sgRNA) target surrounding the stop codon region of the *vmhc* gene and assessed its cleavage efficiency of double-stand breaks (DSBs) induced by the CRISPR/cas9 system (Figure 1A). After optimized concentration of sgRNA and Cas9 protein, we obtained an 80% cleavage efficiency of DSBs in most of the injected embryos (data not shown). Next, we constructed the *vmhc-P2A-EGFP* donor vector that harbors a left homologous arm of ∼2.2 kb DNA spanning the last exon (exon 37) and exon 32 of the *vmhc* gene, *P2A-EGFP*, and a right homologous arm of ∼1.9 kb DNA of the *vmhc* 3-UTR sequences downstream of the guide RNA target site (Figure 1A). Notably, the P2A nucleotides encode a short peptide that can cleave two neighboring proteins, enabling induction of polygenes co-expression.

**FIGURE 2:**
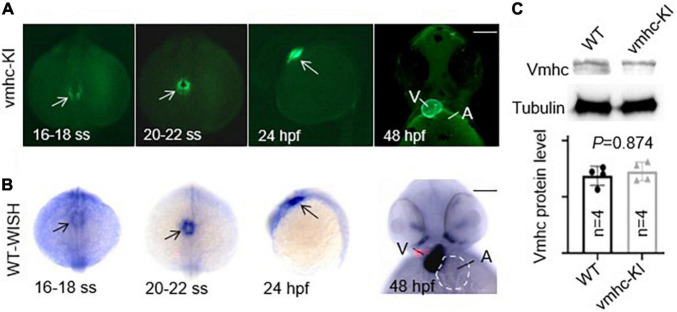
Enhanced green fluorescent protein in the *vmhc^KI–EGFP^* zebrafish KI line recapitulates endogenous expression patterns of the *vmhc* gene. **(A)** EGFP patterns in the *vmhc^KI–EGFP^* KI fish line from 16 to 18 somite stage (SS) to 48 hpf. Scale bar, 100 μm. White arrows point to specific patterns of the EGFP signal. **(B)** Endogenous expression patterns of the *vmhc* gene from 16 to 18 somite stage (SS) to 48 hpf revealed by WISH. Black arrows point to the specific expression pattern of *vmhc* endogenous transcripts. The dotted line outlines the shape of the atrium. Scale bar, 100 μm. **(C)** Western blotting and quantification analysis of the Vmhc protein expression in the *vmhc^KI–EGFP^* KI fish line compared to wild-type (WT) control. *N* = 4. Unpaired 2-tailed Student’s *t*-test. V, ventricle. A, atrium.

After the correct construction of the recombinant *vmhc-P2A-EGFP* donor vector was confirmed by PCR and Sanger sequencing (data not shown), we then co-injected it with the sgRNA/Cas9 complex into one-cell stage embryos. Embryos with a low-mosaic EGFP signal detected in the heart ventricle were raised up and termed as candidate *vmhc^KI–EGFP^* F0 founders. After they reached adulthood, we outcrossed these candidate’s *vmhc^KI–EGFP^* F0 founders to identify stable F1 mutants. Out of 8 F0 founder fish screened, we successfully identified one with stable EGFP signal detected in the F1 offsprings, and the P2A-EGFP sequences knocked in the *vmhc* C-terminal locus was confirmed by PCR and Sanger sequencing (Figure 1B). The EGFP signal was mostly noted in the lateral plate mesoderm, cardiac tube, and ventricle (Figure 2A), which was very similar to the endogenous expression patterns of the *vmhc* gene at corresponding stages detected by WISH (Figure 2B). To determine whether the P2A-EGFP element knocked in the *vmhc* C-terminus would interfere expression of the endogenous vmhc protein, we performed Western blotting analysis and detected no difference in terms of the vmhc protein expression level between the *vmhc^KI–EGFP^* KI fish and wide-type (WT) controls (Figure 2C), suggesting the P2A-EGFP KI event did not interfere expression of the endogenous vmhc protein. Taken together, these results demonstrated that we successfully generated a novel EGFP KI fish line in which the EGFP could faithfully report the spatial and temporal expression of the endogenous *vmhc* gene.

### Compound Paracetamol and Amantadine Hydrochloride Tablets Exposure Caused Cardiac-Looping Defects Dose-Dependently

**FIGURE 3:**
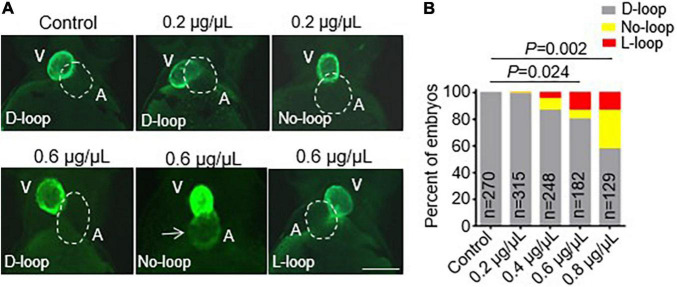
CPAAHTs exposure induced ectopic expression of *vmhc* gene in the atrium and caused cardiac-looping defects to dose-dependently. **(A,B)** Representative images of heart-looping morphology **(A)** and quantification analysis **(B)** of the percentage of the *vmhc^KI–EGFP^* KI embryos with corresponding cardiac-looping structure after exposure to different concentrations of CPAAHTs compared to untreated control at 48 hpf. The arrow points to the ectopic expression of the EGFP signal in the atrium. The dotted line outlines the shape of the atrium. V, ventricle. A, atrium. *N* = 129–315, chi-squared test.

After we successfully generated the *vmhc^KI–EGFP^* KI fish line, we sought to employ it to study the potential toxic effects of CPAAHTs during early cardiogenesis *in vivo*. We found that exposure to CPAAHTs induced heart malformation in a dose-dependent manner. As shown in Figure 3A, all untreated *vmhc^KI–EGFP^* control embryos exhibited exclusively D-loop patterning at 48 hpf. In contrast, embryos treated with 0.2 μg/μl of CPAAHTs started to show L-loop patterning in a small percentage (Figures 3A,B). As the doses were increased to 0.6 or 0.8 μg/μl, respectively, the percentage of embryos with L-loop and no-loop increased significantly, concurrent with apparent cardiac malformation phenotypes. In addition to the abnormal cardiac-looping defects, we also detected ectopic EGFP signal in the atrium of CPAAHTs treated embryos (Figure 3A), suggesting intrinsic factors that defined the ventricle-specified expression of *vmhc* gene were altered. Together, these results showed that CPAAHTs could cause cardiac-looping or LR asymmetry defects during early cardiogenesis.

### Compound Paracetamol and Amantadine Hydrochloride Tablets Exposure Suppressed Expression of Genes Required for Myocardium Differentiation and Left–Right Asymmetry

**FIGURE 4:**
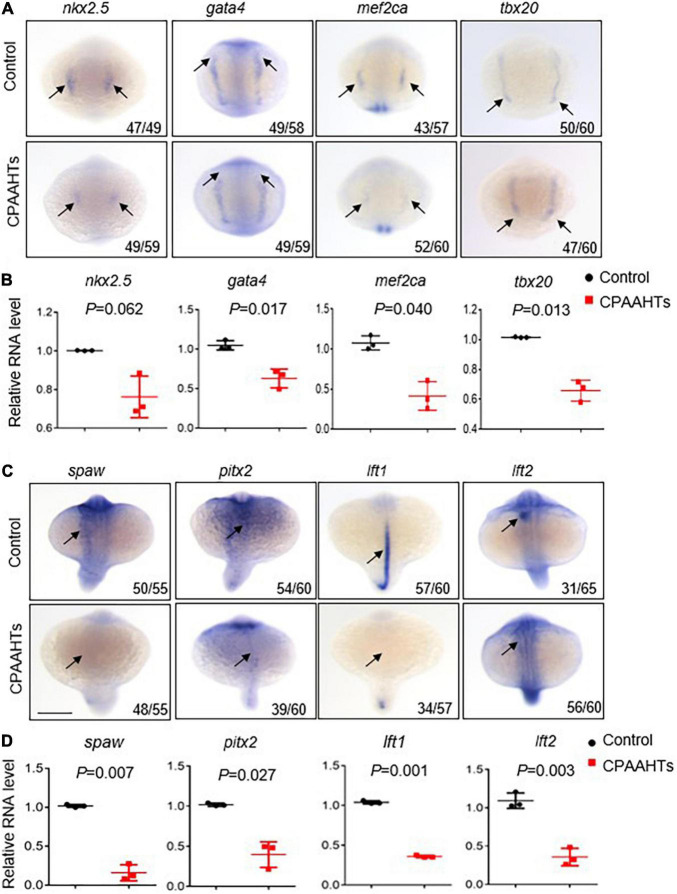
CPAAHTs exposure suppressed the expression of genes required for myocardium differentiation and LR asymmetry. **(A,B)** Dorsal view of WISH images **(A)** and quantitative RT-PCR analysis **(B)** of transcription factors *nkx2.5, gata4, mef2ca*, and *tbx20* in 0.8 μg/μl CPAAHTs treated embryos compared with untreated controls at 8-somite stage (SS). Arrows point to expected expression patterns for the corresponding genes. *N* = 3. Unpaired 2-tailed Student’s *t*-test. **(C,D)** Dorsal view of WISH images **(C)** and quantitative RT-PCR analysis **(D)** of Nodal-related gene *spaw* and its downstream target genes in 0.8 μg/μl CPAAHTs treated embryos compared with untreated controls at 25- to 28-SS. Arrows point to the expected expression patterns for the corresponding genes. *N* = 3. Unpaired 2-tailed Student’s *t*-test.

To investigate molecular mechanisms underlying the CPAAHTs-induced ectopic expression of *vmhc* gene in the atrium, we first analyzed expression patterns of several key transcription factors including *nkx2.5*, *gata4*, *mef2ca*, and *tbx20* that define the myocardial precursor cell differentiation during early cardiac development. Our WISH results showed that, in untreated control embryos, these 4 genes were mostly expressed as bilateral stripes in the heart-forming region at 8-somite stage (Figure 4A). In contrast, CPAAHTs-treated embryos showed a dramatic reduction in expression of all 4 genes. Furthermore, we performed quantitative RT-PCR analysis and confirmed significant transcriptional reduction for *gata4, mef2ca*, and *tbx20* genes, with marginally decreased expression of *nkx2.5* detected as well (Figure 4B). Thus, it is plausible to speculate that CPAAHTs might cause cardiac developmental defects by disturbing the differentiation of myocardial precursor cells. Given that embryos exposed to CPAAHTs exhibited obvious cardiac-looping or LR asymmetry defects, we hypothesized that CPAAHTs might disrupt early cardiac development through inhibiting gene expression of key genes specifying cardiac LR asymmetry. We particularly examined the expression of *spaw*, a key factor of the Nodal signaling pathway that plays a critical role in LR patterning, and its downstream target genes including *pitx2, lft1*, and *lft2.* The WISH results demonstrated that near-complete loss of the *spaw* expression was observed in the CPAAHTs-treated embryos, in contrast to its robust expression in the untreated control embryos (Figure 4C). Consistently, the expression levels of other *spaw* downstream genes such as *pitx2*, *lft1*, and *lft2* were also reduced drastically. Similarly, quantitative RT-PCR was carried out and confirmed the significantly decreased expression of these 4 genes in the CPAAHTs-treated embryos as well (Figure 4D). Together, these results supported our hypothesis that CPAAHTs exposure caused LR asymmetry defects at least partially through suppressing the expression of key factors of the Nodal signaling pathway.

## Discussion

Congenital heart disease is a form of birth defect representing the most common congenital anomaly in newborns (1, 18). CHD could be caused by misuse of some drugs during pregnancy. Amantadine hydrochloride is an antagonist of the *N*-methyl-D-aspartate receptor used to treat influenza A virus, Parkinson’s disease, and dyskinesia. However, cases of acute cardiotoxicity manifested by cardiac arrhythmia such as right ventricular outflow tract tachycardia and QT elongation had been reported in patients after amantadine overdose (19–21). In addition, cases of newborns with complex cardiovascular lesions and ToF and tibial hemimelia associated with maternal exposure to amantadine were also reported (6). Despite the clinical ramifications of amantadine treatment, limited studies using animal models to evaluate the potential side effects of overdose amantadine on early cardiac development and the related etiologic mechanisms have been reported.

Here, we took advantage of the zebrafish as a prolific animal model for studying amantadine drug effects and pathogenesis on early cardiac development *in vivo*. We utilized the feature of HR pathway triggered by the CRISPR/Cas9 system that enables more precise targeted insertion of exogenous sequences (22), and precisely knocked in the *P2A-EGFP* exogenous sequences into the C-terminal of the target gene *vmhc* in zebrafish. Comparison between the expression dynamic of the endogenous *vmhc* transcripts revealed by WISH and the EGFP patterns in the so-called *vmhc^KI–EGFP^* KI line showed the same spatial and temporal expression dynamics. Together with our Western blot analysis data to show the level of vmhc protein was not changed in the *vmhc^KI–EGFP^* KI line, these compelling results demonstrated that we successfully generated a novel zebrafish KI line in which the EGFP reporter could faithfully reveal the spatial and temporal expression patterns of endogenous *vmhc* gene, without interfering the expression of its encoded vmhc protein. Different from other more commonly used fluorescence reporter lines to label both ventricle and atrium in zebrafish which were often generated by transgenic techniques, this *vmhc^KI–EGFP^* stable KI reporter line could not only be used to conveniently assess potential sides effects of compound drugs such as CPHAATs on heart morphology changes during early cardiogenesis, but also has the potential to reflect directly or indirectly the expression dynamics of the endogenous *vmhc* genes. Indeed, this *vmhc^KI–EGFP^* KI model allows us to reveal abnormal cardiac-looping or cardiac LR asymmetry defects caused by CPAAHTs exposure dose-dependently. Exposure to CPAAHTs at a concentration of 0.6 or 0.8 μg/μl, but not 0.4 or 0.2 μg/μl, disrupted normal heart development and caused significant heart-looping defects. Notably, these in-water doses employed here were determined experimentally, as no practical dose conversion formula between zebrafish embryos and humans has been established yet (23). In addition to the heart-looping defects, intriguingly, this *vmhc^KI–EGFP^* KI model also allows us to detect EGFP signal in the atrium after CPAAHTs exposure, indicating ectopic expression of otherwise ventricle-restrictive expression of the *vmhc* gene. These data suggest the regulatory networks that determine the atrial and ventricular chamber-specific expression of *vmhc* were disturbed by CPAAHTs drug exposure.

In zebrafish, the heart is the first organ to form and function during embryogenesis (24). During heart development of zebrafish, many key transcription factors orchestrate this process. For example, *nkx2.5*, the earliest marker of myocardial precursor cells, plays an essential role in determining the morphology of ventricle and atrium during cardiac progenitor cells differentiation (25–28). While other transcription factors such as *gata4* (29), *mef2ca* (30), and *tbx20* (31) form a key regulatory network indispensable to maintain the normal cardiac development. In addition, the heart is one of the earliest internal LR asymmetric organs with conserved regulatory mechanism across different species (32, 33). For example, the nodal pathway plays a conserved role in LR asymmetric morphogenesis through establishing initial molecular differences. Furthermore, the nodal-related gene *southpaw* (*spaw*), and its downstream target genes such as *pitx2*, *lefty1* (*lft1*), and *lefty2* (*lft2*) are crucial to regulate LR asymmetric development (33–36). In our newly established model, we explored the molecular mechanism underlying the cardiac developmental defect and ectopic expression of the *vmhc* gene in the atrial caused by CPAAHTs exposure, through analyzing the expression of these transcription factors governing the myocardial precursor cells differentiation and LR asymmetrical development. Based on our WISH and quantitative RT-PCR results, we found expression for most of the previously described transcription factors, and their corresponding downstream targets were either significantly reduced or near lost in the CPAAHTs exposure group compared with the controls. These results indicated that overdose of CPAAHTs might cause LR asymmetry defects by suppressing the expression of key transcription factors such as *nkx2.5* and *gata4* which are required to specify the myocardium differentiation and the Nodal signaling pathway that is required to establish the early LR asymmetry.

In summary, we generated a novel zebrafish KI stable line in which the EGFP reporter could faithfully recapitulate the endogenous expression dynamics of the ventricle chamber-specific expression of *vmhc* gene. This EGFP KI stable line was successfully employed to evaluate the potential side effects of CPAAHTs on early cardiac development and malformation in the zebrafish animal model. Mechanistically, the CPAAHTs-induced cardiac malformation could likely be conveyed by suppressing the expression of key genes required for cardiac myocardium differentiation and LR asymmetry. Furthermore, the application of this novel zebrafish EGFP KI reporter line generated here could potentially be extended to assess safety for other drugs during early cardiogenesis as well.

## Data Availability Statement

The original contributions presented in the study are included in the article/supplementary material, further inquiries can be directed to the corresponding authors.

## Ethics Statement

The animal study was reviewed and approved by the Institutional Animal Care and Use Committee (IACUC), Hunan Normal University. Written informed consent was obtained from the owners for the participation of their animals in this study.

## Author Contributions

SO, W-MQ, and YD contributed to the conception and design of the study. SO and Y-JN carried out the experiments, performed the statistical analysis, and wrote the first draft of the manuscript. YD and Y-HD supervised the study and edited the manuscript. All authors read and approved the submission of the manuscript.

## Conflict of Interest

The authors declare that the research was conducted in the absence of any commercial or financial relationships that could be construed as a potential conflict of interest.

## Publisher’s Note

All claims expressed in this article are solely those of the authors and do not necessarily represent those of their affiliated organizations, or those of the publisher, the editors and the reviewers. Any product that may be evaluated in this article, or claim that may be made by its manufacturer, is not guaranteed or endorsed by the publisher.
